# The interplay between heart rate variability, inflammation, and lipid accumulation: Implications for cardiometabolic risk

**DOI:** 10.14814/phy2.70313

**Published:** 2025-04-26

**Authors:** Cameron R. Wiley, Vida Pourmand, Sarah K. Stevens, Marc N. Jarczok, Joachim E. Fischer, Dario Boschiero, Eleonora Poggiogalle, Julian Koenig, Julian F. Thayer, DeWayne P. Williams

**Affiliations:** ^1^ Department of Psychological Science University of California, Irvine Irvine California USA; ^2^ Institute of Medical Psychology, Center for Psychosocial Medicine University Hospital Heidelberg, Ruprecht‐Karls‐University Heidelberg Heidelberg Germany; ^3^ Department of Psychosomatic Medicine and Psychotherapy Ulm University Medical Center Ulm Germany; ^4^ Mannheim Institute of Public Health, Social and Preventive Medicine Mannheim Medical Faculty, Heidelberg University Mannheim Germany; ^5^ BIOTEKNA Biomedical Technologies Venice Italy; ^6^ Open Academy of Medicine London UK; ^7^ Department of Experimental Medicine “Sapienza” University of Rome Rome Italy; ^8^ Department of Child and Adolescent Psychiatry, Psychosomatics and Psychotherapy, Faculty of Medicine and University Hospital Cologne University of Cologne Cologne Germany

**Keywords:** body mass index, cardiovascular disease risk, C‐reactive protein, heart rate variability, lipid accumulation product

## Abstract

Deleterious adiposity (e.g., obesity) is considered an inflammatory condition that increases risk for cardiovascular diseases. Lower heart rate variability (HRV), an independent predictor of cardiovascular disease risk, is linked with higher levels of adiposity and inflammation. However, indices of adiposity vary in their strength of association with disease risk. Body mass index (BMI) is a modest predictor of disease, while the lipid accumulation product (LAP) better predicts disease risk. The current investigation used cross‐sectional and prospective designs to probe the differential associations between HRV and multiple measures of adiposity (e.g., LAP and BMI) and examine if inflammation (measured via C‐reactive protein; CRP) mediated these associations. Study 1 showed that HRV was more strongly linked with LAP relative to other adiposity measures and that this link was mediated by CRP. Study 2 replicated Study 1 results and showed that this association remained significant 4 years later. Our novel findings are consistent with studies suggesting LAP may be a superior measure of cardiovascular disease risk relative to other measures of adiposity. Importantly, the strong link between HRV and LAP was mediated by inflammation, highlighting the key role of the cholinergic anti‐inflammatory pathway in regulating obesity and associated health consequences.

## 1 INTRODUCTION

Obesity is considered an inflammatory disorder that serves as a major risk factor for numerous cardiovascular diseases. Body mass index (BMI, kg/m^2^) is a commonly used indicator of relative adiposity and is often cited as a predictor of such diseases (e.g., Khan et al., 2018; McGee & Diverse Populations Collaboration, 2005). However, higher BMI is only modestly associated with higher rates of cardiovascular disease (Bennasar‐Veny et al., 2013; McGee & Diverse Populations Collaboration, 2005). One potential explanation for this modest association is that BMI is an approximate, rather than specific, measure of excess weight that does not consider the differential distribution of adipose tissue across the body (Frayn et al., 2003; Prentice & Jebb, 2001). Given that BMI serves as a nonspecific indicator of adiposity, recent research suggests the lipid accumulation product (LAP) is a more specific indicator of deleterious excess weight and thus, medical risk (Chiang & Koo, 2012; Ioachimescu et al., 2010; Kahn, 2005; Taverna et al., 2011). LAP is measured using both waist circumference (in centimeters) and fasting triglyceride concentration (mmol/L). Waist circumference serves as a measure of intra‐abdominal fat, and triglyceride concentration serves as a measure of an overaccumulation of lipids that circulate in the bloodstream (in the form of triglycerides). Overall, previous research has found LAP to be a better predictor of cardiovascular diseases and other medical risks compared to BMI (Kahn, 2005), highlighting the importance and “specificity” of this measure in the clinical setting.

Both higher BMI (Koenig et al., 2014; Williams et al., 2017) and greater cardiovascular disease risk (see Thayer et al., 2010) are associated with lower vagal activity, which represents a maladaptive physiological health profile (Pavlov & Tracey, 2012; Thayer & Lane, 2000; Thayer & Sternberg, 2006). Vagally mediated heart rate variability (HRV), defined as the rapid beat‐to‐beat fluctuations in heart rate mediated by the vagus nerve, serves as a valid and reliable index of vagal activity (Laborde et al., 2017). Thus, lower HRV is considered a significant and independent risk factor for cardiovascular disease risk and all‐cause mortality (Jarczok et al., 2022; Thayer et al., 2010; Thayer & Sternberg, 2006). Likewise, lower HRV has also been associated with higher BMI (Koenig et al., 2014; Williams et al., 2017). However, research has yet to examine the association between HRV, an important indicator of vagal activity and predictor of health status, and LAP, which is considered to be better at both specifying deleterious adiposity and predicting cardiovascular disease risk compared to BMI (see Kahn, 2005, for review).

The inflammatory reflex, or the innate immune responses that take place during pathogen invasion and tissue injury (Pavlov & Tracey, 2012; Tracey, 2002), has also been implicated in the etiology of both deleterious adiposity (i.e., obesity) and cardiovascular disease risk (Berg & Scherer, 2005; Libby, 2006). More specifically, C‐reactive protein (CRP, mmol/L) has been identified as a common marker of systemic inflammation that underlies several health risks and outcomes and has been linked with an overall increased risk of cardiovascular diseases (Fonseca & Izar, 2016; Lagrand et al., 1999). Studies have highlighted that higher CRP is associated with higher BMI (Visser et al., 1999); see Chooi et al., 2019 for review), higher LAP (Mirmiran et al., 2014), and lower HRV (see Williams et al., 2019 for review). While the vagus nerve has been established as a crucial part of the inflammatory response (Pavlov et al., 2003; Pavlov & Tracey, 2012) and lower HRV has been associated with higher CRP (Jarczok et al., 2014; Sajadieh et al., 2004), studies have yet to explore links between HRV, CRP, and LAP (as opposed to BMI), which is warranted to better understand the development of adiposity and cardiovascular disease risk.

### 1.1 Current studies

The current investigation sought to explore the interplay between adiposity, inflammation, and cardiovascular function via two studies. Study 1, to our knowledge, is the first to cross‐sectionally investigate and compare associations between 24‐hour HRV and various measures of adiposity, including LAP, waist circumference, triglyceride concentration, and BMI. Given previous evidence demonstrating a bidirectional link between autonomic and inflammatory functioning, as well as growing evidence showing that inflammation is strongly related to both adiposity and cardiovascular functioning, Study 1 used archival data to determine if inflammation, indexed using CRP, mediated the link between HRV and the adiposity measure(s) (e.g., BMI and LAP) that yielded the strongest association cross‐sectionally.

Study 2 sought to determine if inflammation, indexed using CRP, mediated the link between HRV and adiposity prospectively over the course of four  years. We hypothesized that HRV would be significantly and negatively associated with all adiposity measures, while highlighting a novel association between HRV and LAP. As HRV is an independent predictor of cardiovascular disease risk (Thayer et al., 2010), and LAP is superior to BMI in predicting such risk (Kahn, 2005), we hypothesized that HRV should be more strongly associated with LAP relative to other measures of adiposity. We also hypothesized that CRP would be positively associated with all adiposity measures and negatively associated with HRV.

## 2 MATERIALS AND METHODS

### 2.1 Study 1 materials and methods

Study 1 used cross‐sectional data collected from September 2003 to February 2004, which included a sample of healthy employees from an aviation company that participated in a voluntary on‐site health assessment. Questionnaire data were collected, and medical examinations were also carried out during this visit. The study was approved by the Institutional Review Board of the Federal Institute of Technology, Zurich, Switzerland, and all participants provided written informed consent prior to their assessment. Data from a total of 641 individuals (70 females, *M*_Age_ = 42 years, *SD*_Age_ = 11 years) were available for analyses.

#### 2.1.1 Procedures and measures

Continuous inter‐beat intervals were collected using a Mini‐Vitaport electrocardiogram logger (Becker Medical Systems, Karlsruhe, Germany) at a sampling rate of 400 Hz. Participants were attached with ambulatory electrocardiogram devices between 9 and 12 am. Once attached, individuals proceeded with their day, and between 7:15 and 8:00 am on the following day, the ambulatory electrocardiogram was disconnected. Inter‐beat intervals were calculated as the time between successive R‐spikes. Intervals that corresponded to a mean heart rate (HR) <30 or >200 as well as changes of over 30% were excluded (i.e., artifact correction). The root mean square of successive differences (RMSSD) is a reliable, stable, and valid time‐domain index of vagal activity (Task Force, 1996) and was used as the primary index of HRV in Study 1. RMSSD was calculated from the average of all valid adjacent beat‐to‐beat intervals during the entire recording period in 5 min blocks.

The morning after HRV data was collected, CRP was collected via fasting blood samples (between 6:30 and 8:30 am) and immediately transported to the commercial laboratory Synlab (Augsburg) where values were obtained using a high‐sensitivity assay (Dade Behring, Schwalbach, Germany; Synlab, Augsburg, Germany). On the same day as the CRP collection, all participants underwent an additional fasting blood draw between 9 and 11 am to assess triglyceride concentration (TRI; mmol/L), which was followed by a medical examination. During this time, subjects' body measures (weight in kg, height in cm, hip circumference in cm, and waist circumference [WC] in cm) were taken. BMI was obtained per common calculation using height and weight (kg/m^2^). LAP was calculated using the following formulas proposed by Kahn (2005):

$$\begin{array}{c} \mathrm{LAP}\;\text{for females}=\left(\text{waist circumference}\;\left[\mathrm{cm}\right]–58\right)\\ \times \left(\text{triglyceride concentration}\;\left[\text{mmol}/L\right]\right) \end{array}$$

$$\begin{array}{c} \mathrm{LAP}\;\text{for males}=\left(\text{waist circumference}\;\left[\mathrm{cm}\right]–65\right)\\ \times \left(\text{triglyceride concentration}\;\left[\text{mmol}/L\right]\right) \end{array}$$

### 2.2 Study 2 materials and methods

#### 2.2.1 Procedures and measures

Healthy employees of an industrial company in Southern Germany participated in a voluntary on‐site health assessment during regular work hours in 2007 (time 1|T1), with a follow‐up assessment in 2011 (time 2|T2). The sample had an age range of 18–65 years and spanned all levels of socioeconomic status. The study was approved by the Medical Ethic Committee II of the Medical Faculty Mannheim of Heidelberg University (approval numbers 2007‐009E‐MA and 2010‐296E‐MA). All participants gave written informed consent prior to examination. Data from a total of 227 individuals (21 females, *M*_Age_ = 48 years, *SD*_Age_ = 8 years) were available for analyses.

HRV was obtained using wet electrodes (Ambu BluSensor, Ølstykke, Denmark) connected to a five‐lead Cardio‐Scout Holder electrocardiogram system (SR‐Medizinelektronik, Stuttgart, Germany; sampling rate 500 Hz). Ambulatory electrocardiogram devices were attached to participants in the morning and then proceeded with their day until the next morning, when the ambulatory electrocardiogram was disconnected. Using Task Force (1996) guidelines, researchers at the Center for Neuropsychological Research (University of Trier, Trier, Germany) analyzed the raw electrocardiogram recordings. High‐frequency HRV (i.e., HF‐HRV; 0.15 Hz‐0.40 Hz) is a reliable, stable, and valid frequency‐domain index of vagal activity (Task Force, 1996) and was used as the primary index of HRV in Study 2. HRV measures were only available at T1. It is critical to note that we utilize HF‐HRV primarily in Study 2 to show consistency across vagally mediated HRV metrics, and the association between RMSSD‐HRV and HF‐HRV in these data are highly correlated as expected (*r* = 0.81).

The morning after HRV data were collected, CRP was obtained via fasting blood samples (collected between 6:30 and 8:30 am). Blood samples were taken at T1 to a commercial laboratory (Synlab, Augsburg, Germany), and CRP was indexed via a high‐sensitivity assay (Dade Behring, Schwalbach, Germany). For T2, the measurement protocols were the same, with the exception of the health risk assessment and data collection being conducted by an agent (HealthVision Ltd., Berlingen, Switzerland). CRP was indexed at T2 using a high‐sensitivity assay (OSR6199; Beckman Coulter GmbH, Krefeld, Germany).

### 2.3 Statistical analyses

Statistical tests were conducted using IBM SPSS Statistics (ver. 27, IBM Chicago, IL, USA). HRV (i.e., RMSSD) and all adiposity values were skewed and successfully log‐transformed to meet assumptions of linear analyses. Means and standard deviations presented are raw (untransformed) values. First, zero‐order (Pearson's *r*) correlation coefficients were conducted to determine associations between HRV (including each period of the day) and measures of adiposity. Both age and sex have been shown to influence both HRV (Choi et al., 2006; Koenig & Thayer, 2016) and measures of adiposity (Cooper et al., 2021; Gallagher et al., 1996), and thus we also conducted partial *r* correlations that adjusted for these variables. We used StatSoft Statistica 6.0 (StatSoft, Inc., Tulsa, OK) to determine hypothesized differences between correlation coefficients using Fisher's *r*‐to‐*z* transformation. All tests were two‐tailed with significance set at alpha of 0.05, with the exception of Fisher's *r*‐to‐*z* transformation correlation coefficient differences tests, which were conducted using one‐tailed tests given that our directional hypotheses are well supported by the literature.

For mediation analyses in both studies, an SPSS custom dialog called PROCESS (Hayes, 2012) was used to examine how HRV, CRP, and measures of adiposity (BMI, LAP, TRI, WC) were linked. For Study 1, we specified “Model 4” which allowed us to specify an independent variable, a mediating variable, and a dependent variable. For Study 2, we selected “Model 6” which allowed us to specify an independent variable, multiple mediating variables, and a dependent variable. Age and sex were entered as covariates in all mediation models. 95% bootstrapped confidence intervals (95% CI; 5000 samples) were used to determine the significance of each mediating or indirect effect (Hayes, 2012; see MacKinnon et al., 2004 for details regarding the bootstrapping procedure). Statistics reported include unstandardized betas (B), standard error (in brackets), and the bootstrapped CI's (lower limit, upper limit) for each path of the model.

## 3 RESULTS

### 3.1 Study 1 results

Descriptive statistics for the full sample, including variables of interest, are presented in Table 1. Correlation results showed HRV to be significantly negatively associated with BMI (*r* = −0.134, 95% CI [−0.21, −0.06], *p =* 0.001), WC (*r* = −0.160, 95% CI [−0.23, −0.08], *p <* 0.001), TRI (*r* = −0.163, 95% CI [−0.24, −0.09], *p =* 0.006), and LAP (*r* = −0.205, 95% CI [−0.29, −0.13], *p <* 0.001). One‐tailed Fisher's *r*‐to‐*z* transformation tests showed that the correlation between HRV and LAP was not significantly greater than that of HRV and BMI (*z* = −1.31, *p =* 0.095), TRI (*z* = −0.78; *p =* 0.218), or WC (*z* = −0.83; *p =* 0.203). HR was not significantly associated with any index of adiposity (each *p* > 0.164).

**TABLE 1:** Descriptive statistics for Study 1.

Variable	Mean (SD)
Age	41.56 (11.48)
Height	179.16 (7.69)
Weight	84.61 (13.60)
BMI	26.31 (3.78)
WC	91.98 (10.57)
HC	100.63 (7.12)
WC/HC	0.91 (0.07)
TRI	154.07 (126.18)
LAP	60.60 (63.06)
HR	83.17 (12.03)
HRV	38.99 (14.56)
CRP	0.20 (0.38)

*Note*: This table represents means and standard deviations (SD) of all variables. Age values represent years, height was measured in centimeters (cm), BMI represents body mass index (kg/m^2^), WC represents waist circumference, and CRP represents C‐reactive protein (mmol/L). HR represents heart rate (beats per minute). LAP represents the lipid accumulation product (mol/cm^3^). Vagally mediated heart rate variability (HRV) is represented as the log‐transformed root mean square of successive differences (RMSSD; ms).

Partial *r* correlation results adjusting for sex and age showed a significant and negative association between HRV and both TRI (*r*_partial_ = −0.122, 95% CI [−0.20, −0.05], *p =* 0.004) and LAP (*r*_partial_ = −0.134, 95% CI [−0.21, −0.06], *p =* 0.002), but associations were attenuated for BMI (*r*_partial_ = −0.084, 95% CI [−0.16, −0.01], *p =* 0.054) and WC (*r*_partial_ = −0.080, 95% CI [−0.16, −0.003], *p =* 0.066). Results also showed a negative association between CRP and HRV (*r*_partial_ = −0.146, 95% CI [−0.22, −0.07], *p <* 0.001), and positive associations between CRP and all other measures of interest including BMI (*r*_partial_ = 0.375, 95% CI [0.31, 0.44], *p <* 0.001), WC (*r*_partial_ = 0.379, 95% CI [0.31, 0.44], *p <* 0.001), TRI (*r*_partial_ = 0.279, 95% CI [0.21, 0.35], *p <* 0.001), LAP (*r*_partial_ = 0.370, 95% CI [0.30, 0.43], *p <* 0.001), and HR (*r*_partial_ = 0.186, 95% CI [0.11, 0.26] *p <* 0.001). HR was only significantly related to LAP (*r*_partial_ = 0.105, *p <* 0.05). One‐tailed Fisher's *r*‐to‐*z* transformation tests showed that the correlation between HRV and LAP was not significantly higher than that of HRV and BMI (*z* = 0.90, *p =* 0.184), TRI (*z* = −0.22; *p =* 0.413), or WC (*z* = −0.98; *p =* 0.164). The association between CRP and TRI was statistically significant, yet weaker relative to link between CRP and BMI, WC, and LAP (each *p* < 0.05). All Pearson's *r* and partial *r* correlation coefficients are presented in Table 2.

**TABLE 2:** Zero‐order and partial correlations between variables of interest for study.

Variable	1	2	3	4	5	6	7
1. BMI	–	0.847**	0.366**	0.646**	0.091	−0.084	0.375**
2. WC	0.857**	–	0.367**	0.724**	0.095	−0.080	0.379**
3. TRI	0.410**	0.407*	–	0.899**	0.088	−0.122**	0.279**
4. LAP	0.690**	0.748**	0.764**	–	0.105*	−0.134**	0.370**
5. HR	−0.021	−0.061	0.015	−0.004	–	−0.318**	0.186**
6. HRV	−0.134**	−0.160**	−0.163**	−0.205**	0.250**	–	−0.146**
7. CRP	0.335**	0.272**	0.262**	0.332**	0.177**	−0.192**	–

*Note*: This table represents zero‐order (Pearson's *r*; below the diagonal) and partial *r* (statistically adjusting for age and sex; above the diagonal) correlation coefficients between HRV, indices of adiposity, and inflammation. BMI represents body mass index (kg/m^2^), waist circumference (WC) is measured in cm, TRI represents triglyceride fasting concentration (mmol/L), LAP represents the lipid accumulation product (mol/cm^3^), TRI represents triglyceride fasting concentration (mg/dl), HR represents heart rate (beats per minute), and CRP represents C‐reactive protein (mmol/L). Vagally mediated heart rate variability (HRV) is represented as the log‐transformed high‐frequency heart rate variability values (ms^2^). Bolded *p* values represent significant correlation coefficients (*p < 0.05, **p < 0.01).

**FIGURE 1:**
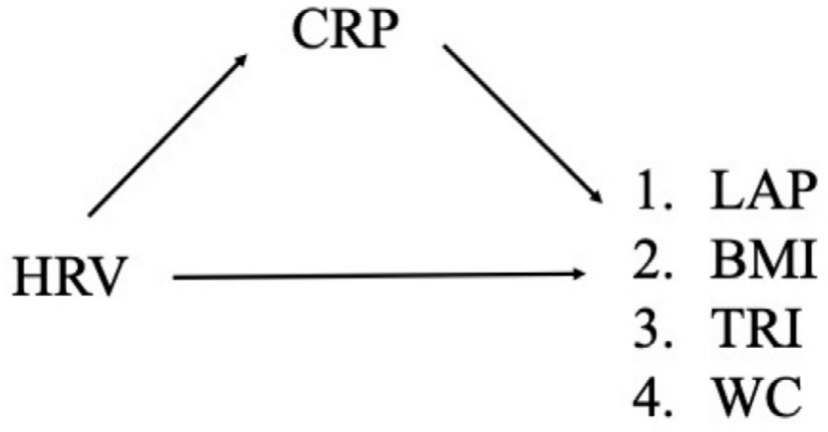
Mediation model paths for Study 1. Study 1 tested the effect of 24‐h heart rate variability [HRV] on adiposity measures (lipid accumulation product [LAP], body mass index [BMI], triglyceride fasting concentration [TRI], waist circumference [WC]), mediated by C‐reactive protein [CRP]. The final model used was Model 1 in the figure above. We tested alternative models of the final model by switching around the X, M, and Y, resulting in five alternative models.

CRP mediated the association between HRV and all measures of adiposity, including LAP (*B* = −0.13, SE = 0.04, 95% CI_boot_ [−0.20, −0.06]), BMI (*B* = −0.02, SE = 0.01, 95% CI_boot_ [−0.04, −0.01]), TRI (*B* = −0.07, SE = 0.02, 95% CI_boot_ [−0.11, −0.03]), and WC (*B* = −0.02, SE = 0.005, 95% CI_boot_ [−0.03, −0.01]). Analysis of the confidence intervals indicated that the indirect effect appeared strongest between HRV and LAP (95% CI_boot_ [−0.20, −0.06]), followed by HRV and TRI (95% CI_boot_ [−0.11, −0.03]), accompanied by a weaker and narrower CI for the association between HRV and both BMI (95% CI_boot_ [−0.04, −0.01]) and WC (95% CI_boot_ [−0.03, −0.01]). See Figure 1 for a conceptual model of the mediation analyses for Study 1.

In sum, while CRP mediated the link between HRV and all adiposity parameters, the size of the effect was largest for the indirect link between HRV and LAP.

### 3.2 Study 2 results

Descriptive statistics for the full sample, including variables of interest, are presented in Table 3. Correlation results showed HRV to be significantly and negatively associated with T1 LAP (*r* = −0.274, 95% CI [−0.39, −0.15], *p <* 0.01) and T1 CRP (*r* = −0.130, 95% CI [−0.26, −0.01], *p* = 0.04) as well as T2 LAP (*r* = −0.213, 95% CI [−0.33, −0.09], *p* < 0.01), but the effect was slightly attenuated for T2 CRP (*r* = −0.122, 95% CI[−0.25, 0.01], *p =* 0.06). LAP at both timepoints was significantly and positively associated with CRP at both timepoints (each *p* > 0.05). Lower T1 HRV was also linked with higher T1 CRP (*r* = −0.194, 95% CI [−0.32, −0.05], *p =* 0.003) and higher T2 CRP (*r* = −0.134, 95% CI [−0.24, −0.03], *p =* 0.040). One‐tailed Fisher's *r*‐to‐*z* transformation tests showed that the correlation between T1 HRV and T1 LAP (*r* = −0.274) was trending to be higher than that of T1 HRV and T1 BMI (*r* = −0.135; *z* = −1.29, *p* = 0.099), but not for T1 TRI (*z* = −0.163; *p =* 0.212) or T1 WC (*z* = −0.96; *p =* 0.169).

**TABLE 3:** Descriptive statistics for Study 2.

Variable	Mean (SD)
Age (T2)	47.77 (8.23)
Height (T2)	177.72 (7.67)
Weight (T1)	84.35 (13.21)
BMI (T1)	24.52 (3.28)
BMI (T2)	25.12 (4.13)
WC (T1)	95.14 (10.79)
WC (T2)	96.64 (11.60)
TRI (T1)	137.97 (83.55)
TRI (T2)	129.63 (73.56)
LAP (T1)	57.83 (42.63)
LAP (T2)	56.14 (38.64)
HRV (T1)	5.38 (0.83)
HR (T1)	66.23 (10.58)
CRP (T1)	1.83 (1.77)
CRP (T2)	0.16 (0.20)

*Note*: This table represents means and standard deviations (SD) of all variables. Age values represent years, height was measured in centimeters (cm), BMI represents body mass index (kg/m^2^); waist circumference (WC) is measured in cm, TRI represents triglyceride fasting concentration (mg/dl), LAP represents the lipid accumulation product (mol/cm^3^), CRP represents C‐reactive protein (mmol/L), and HR (beats per minute) represents. Vagally mediated heart rate variability (HRV) is represented as the log‐transformed high‐frequency (ms^2^) heart rate variability values; T1 indicates time 1 and T2 indicates time 2.

Partial *r* correlation results indicated a statistically significant negative association between T1 HRV and LAP at both time points. However, HRV was significantly and negatively associated with T1 BMI but was not significantly associated with T2 BMI, although the latter association was in the hypothesized negative direction. One‐tailed Fisher's *r*‐to‐*z* transformation tests showed that the correlation between T1 HRV and T1 LAP was higher than that of T1 HRV and T1 BMI (*z* = −1.54, *p* = 0.061). T1 HRV was negatively associated with T1 CRP (*r*_partial_ = −0.174, 95% CI [−0.30, −0.05], *p =* 0.008) and T2 CRP (*r*_partial_ = −0.121, 95% CI [−0.25, 0.01], *p =* 0.065). Results also showed that there was a positive association between LAP and CRP at both time points (each *p* < 0.05). See Table 4 for all correlation results.

**TABLE 4:** Zero‐order and partial correlations between variables of interest for Study 2.

Variable	1	2	3	4	5	6	7	8	9	10	11	12
1. BMI (T1)	–	0.740**	0.631**	0.528**	0.207*	0.157*	0.515**	0.437**	−0.081	0.051	0.224**	0.216**
2. BMI (T2)	0.803**	–	0.475**	0.489**	0.061	0.191*	0.300**	0.441**	−0.066	−0.020	0.166**	0.248**
3. WC (T1)	0.559**	0.416**	–	0.633**	0.289**	0.278**	0.507**	0.473**	−0.162	0.142*	0.319**	0.286**
4. WC (T2)	0.497**	0.462**	0.632**	–	0.190*	0.305**	0.376**	0.508**	−0.129	0.098	0.284**	0.368**
5. TRI (T1)	0.262**	0.142*	0.299**	0.227**	–	0.680**	0.899**	0.577**	−0.194	0.261**	0.170**	0.151**
6. TRI (T2)	0.199**	0.221**	0.288**	0.287**	0.701**	–	0.607**	0.901**	−0.170	0.104	0.231**	0.255**
7. LAP (T1)	0.549**	0.363**	0.518**	0.410**	0.897**	0.631**	–	0.653**	−0.144	0.119	0.308**	0.276**
8. LAP (T2)	0.471**	0.464**	0.471**	0.509**	0.606**	0.895**	0.697**	–	−0.116	0.091	0.269**	0.352**
9. HRV (T1)	−0.152*	−0.081	−0.236**	−0.185**	−0.256**	−0.162*	−0.224**	−0.197**	–	−0.453**	**−0.174**	−0.121
10. HR (T1)	0.025	−0.039	0.109	0.061	0.266**	0.102	0.091	0.057	−0.385**	–	**−0.246**	0.209**
11. CRP (T1)	0.243**	0.215**	0.199**	0.151*	0.128*	0.120	0.216**	0.217**	−0.165*	0.215**	–	0.489
12. CRP (T2)	0.213**	0.253**	0.183**	0.189**	0.103	0.160*	0.183**	0.269**	−0.180*	0.199**	−0.222**	–

*Note*: This table represents zero‐order (Pearson's *r*; below the diagonal) and partial *r* (statistically adjusting for age and sex; above the diagonal) correlation coefficients between HRV, indices of adiposity, and inflammation. BMI represents body mass index (kg/m^2^); waist circumference (WC) is measured in cm; TRI represents triglyceride fasting concentration (mg/dl), LAP represents the lipid accumulation product (mol/cm^3^), and CRP represents C‐reactive protein (mmol/L). Log‐transformed high‐frequency (ms^2^) heart rate variability (HRV) represents baseline HRV at time 1. T1 indicates time 1, and T2 indicates time 2. Values were natural log transformed prior to this analysis (See Methods for details). Bolded *p*‐values represent significant correlation coefficients (*p < 0.05, **p < 0.01).

**FIGURE 2:**
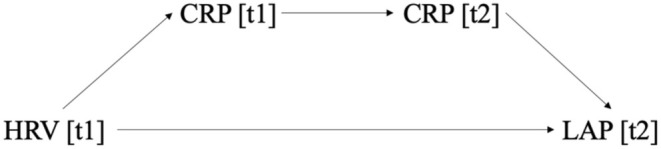
Mediation model path for Study 2. Study 2 tested the effect of 24‐h heart rate variability [HRV] at time 1 [t1] on the lipid accumulation product [LAP] at time 2 [t2], mediated by C‐reactive protein [CRP] at both time 1 and time 2.

Mediation results showed both a direct (*B =* −6.33, SE = 3.24, *p* = 0.05, 95% CI_boot_ [−0.17, −0.13]) and indirect (*B = −*0.41, SE = 0.32, 95% CI_boot_ [−1.24, −0.03]) effect of T1 HRV predicting T2 LAP (Figure 2). First, T1 HRV predicted T1 CRP (*B = −*0.28, SE = 0.15, *p* = 0.057, 95% CI_boot_ [−0.57, 0.01]). Next, T1 CRP predicted T2 CRP (*B =* 0.04, SE = 0.01, *p* < 0.001, 95% CI_boot_ [0.03, 0.06]). Lastly, T2 CRP predicted T2 LAP (*B =* 36.06, SE = 12.91, *p* = 0.01, 95% CI_boot_ [10.61, 61.51]). It is important to note that when including T1 LAP in the model, the indirect effect remained (*B = −*0.08, SE = 0.07, 95% CI_boot_ [−0.26, −0.01]); however, the direct effect was weakened significantly (*B =* −0.61, SE = 2.60, *p* = 0.82, 95% CI_boot_ [−5.72, 4.51]). See Figure 2 for a conceptual model of the mediation analyses for Study 2.

In sum, and adjusting for T1 LAP, the link between T1 HRV and T2 LAP was mediated by CRP at both time points.

## 4 DISCUSSION

### 4.1 General discussion

Both cross‐sectionally and prospectively, this is the first investigation to compare associations between 24‐hour indices of vagally‐mediated HRV and various measures of adiposity—including LAP—in addition to how inflammation (indexed via CRP) mediates these associations. As hypothesized, Study 1 showed RMSSD‐HRV to be associated with all measures of adiposity; however, after adjusting for age and sex, only the association between HRV and LAP remained. This pattern of results was replicated in Study 2 utilizing HF‐HRV. Taken together, these results highlight that relative to other measures of adiposity, especially BMI, LAP appears more strongly linked with indices of vagally‐mediated HRV. Moreover, results from Study 2 showed CRP to mediate the association between HF‐HRV and LAP both cross‐sectionally and 4 years later, suggesting inflammation as a key physiological mechanism linking vagal activity with adiposity (specifically LAP).

### 4.2 Implications

Converging evidence suggests that the overaccumulation of lipids, as indexed by LAP, carries worse cardiovascular consequences than simply having higher body fat, as indexed by BMI (Frayn et al., 2003). Further evidence for this notion is that LAP has been shown to be a better predictor of cardiovascular disease risk compared to BMI (Kahn, 2005; Kyrou et al., 2018). The current data lend novel evidence in this regard, as lower HRV—an independent risk factor for cardiovascular disease—predicts higher LAP. As mentioned, obesity can be described as an inflammatory disease. In the presence of attack or injury to the human body, a reflexive and localized response sets into motion an inflammatory process that alerts the brain to eliminate the pathogenic threat. As such, the inflammatory reflex is known as the body's primary defense against infection, and its malfunction has been implicated in several diseases. The magnitude of the inflammatory response is important, as an extreme or insufficient response can be differentially harmful to the individual (see Tracey, 2009, for a review).

Overall, a core set of physiological structures that involve the vagus nerve is responsible for a quick reflexive action in response to inflammation, known as the cholinergic anti‐inflammatory pathway (Pavlov et al., 2003; Pavlov & Tracey, 2005; Tracey, 2007). The effectiveness of this pathway can be reflected in the longitudinal association of HRV and CRP, such that a dysregulation of the vagus (i.e., lower HRV) may leave the host susceptible to disease via higher circulating pro‐inflammatory cytokines (i.e., higher CRP). Inflammation disrupts the effectiveness of insulin, and this insulin resistance and subsequent glucose and fat accumulation in the liver underpin the link between higher inflammation and obesity, as indexed by higher measures of adiposity (i.e., higher LAP). Our data suggest that this maladaptive physiological profile can exist both cross‐sectionally and longitudinally. In sum, the mechanistic and longitudinal role of CRP linking HRV with LAP is intuitive from theoretical and physiological perspectives (Williams et al., 2019).

As it relates to research on disease risk, adiposity, and physiological function, we stress the importance of including HRV in studies that examine measures of adiposity, especially LAP. Additionally, triglyceride concentration showed a strong and consistent association with HRV relative to both BMI and waist circumference, which replicates evidence from prior investigations (Jarczok et al., 2014; Thayer & Fischer, 2009). This finding is consistent with work that has shown triglyceride concentration to better predict overall disease risk compared to BMI (Bengtsson et al., 1993).

### 4.3 Limitations and future directions

One limitation of the current investigation is that we were underpowered to adequately explore ethnic, sex, and age differences as a plethora of evidence suggests group differences in HRV (e.g., Choi et al., 2006; Hill et al., 2015; Koenig & Thayer, 2016), inflammation (Chung et al., 2019; Klein & Flanagan, 2016), and obesity (Chooi et al., 2019; Cossrow & Falkner, 2004). Importantly, recent work suggests that females and males differ in both mean and the associations between HRV and HR (see Koenig & Thayer, 2016 and Williams et al., 2022 for discussion). Therefore, it is incumbent on future research to carefully consider how these associations may differ as a function of ethnicity, sex, and/or age. In addition, future investigations should be more balanced in terms of sex, as our current sample was predominantly male. Future studies should also standardize analysis methods for biological samples across time points in order to minimize the influence of potential inconsistencies in assay methods. However, the present study focused on the relative associations (rather than the absolute values) of CRP with cardiovascular disease risk factors, and therefore the effect of different assays at the different time points is negligible. It is important that future studies include circulating metabolic metrics such as insulin and glucose. While these data were not present in the current study, such metrics would better highlight how HRV, CRP, and adiposity interact in a complex manner. Another limitation of the current study is that other metrics of inflammation were not available for analysis; future studies should consider additional metrics of inflammation such as Interleukin‐6. Also, future studies should investigate the association between adiposity and HRV at different times of day given the influence of the circadian cycle. Finally, It is important to note that the association between vagal activity (HRV), inflammation (e.g., CRP), and adiposity (e.g., LAP) is not unidirectional. In other words, adiposity can have a detrimental impact on inflammation, which can influence HRV. Thus, future prospective studies should work to understand how inflammation and/or adiposity prospectively influence HRV as a measure of cardiovascular health.

## 5 CONCLUSIONS

In sum, the present studies are the first to show a significant association between HRV and LAP, as well as the mediating effect of inflammation (CRP) on the association of HRV with LAP. Our data lend further support for the idea that LAP may specify deleterious adiposity that can cause cardiovascular complications (Kahn, 2005), as LAP is strongly related to HRV, independent of age and sex. Finally, these data continue to illustrate the importance of assessing and utilizing HRV in clinical studies involving adiposity and health.

## AUTHOR CONTRIBUTIONS

Cameron R. Wiley and Vida Pourmand were involved in formal analysis, data curation, writing—original draft, writing—review and editing, and visualization. Sarah K. Stevens was involved in writing—review and editing. Marc N. Jarczok was involved in conceptualization, methodology, software, validation, investigation, resources, data curation, project administration, funding acquisition, writing—review and editing. Joachim E. Fischer was involved in conceptualization, methodology, investigation, and project administration. Dario Boschiero was involved in conceptualization, methodology, and validation. Eleonora Poggiogalle was involved in methodology and validation. Julian Koenig was involved in conceptualization, writing—review and editing, and supervision. Julian F. Thayer was involved in conceptualization, writing—original draft, writing—review and editing, and supervision. DeWayne P. Williams was involved in conceptualization, formal analysis, data curation, writing—original draft, writing—review and editing, and supervision.

## FUNDING INFORMATION

Core funding was provided from the Mannheim Institute of Public Health for the current research.

## CONFLICT OF INTEREST STATEMENT

Until the end of 2012, Prof. Fischer was CEO and major shareholder of HealthVision Ltd., the company that organized the data collection. The other authors of this study report no relevant conflict of interest with the subject matter or materials discussed or not discussed in the manuscript.

## ETHICS STATEMENT

These studies involving humans were approved by the Institutional Review Board of the Federal Institute of Technology, Zurich, Switzerland and the Medical Ethics Committee II of the Medical Faculty Mannheim of Heidelberg University. The participants provided their written informed consent to participate in these studies.

## Data Availability

The dataset presented in this article, supporting the conclusions of this article, can be made available by the authors upon reasonable request.
